# Phillips on Spermatic Discharges

**Published:** 1848

**Authors:** Benjamin Phillips


### ARTICLE III.

*Spermatic Discharges: their Effects and Treatment.* By BENJAMIN PHILLIPS, Esq., F.R.S.

I have no means of knowing Lallemand's experience at the time he concluded his work; but the cases contained in his treatise amount to 115. My own experience very much exceeds that: I have been consulted in nearly 700 cases. I have memoranda of 623. He gives the particulars of 115 cases of involuntary discharges; of these it is said, 20 were the result of gonorrhœa, 10 of skin diseases, 13 of rectum complaints, 14 of masturbation, 21 of venereal excesses, 21 of various congenital defects of the genital organs. Of these cases he seems to have cured about 90, and to have failed apparently in 8 or 9 cases. He seems to have cured 55 mainly by the use of lunar caustic. He appears to have cured 34 by other means, caustic having failed, or it was not used.

Of the 623 cases (in Mr. Phillips' experience) only 33 had sustained any evil influence than that which was fairly attributable to great mental depression, and that depression was commonly the result, not of the extent of the discharge, but of the anticipated consequences of its continuance—those anticipations being, in most cases, derived from the perusal of such books as are usually circulated on the subject. Of the 623 cases 581 were under 25 years of age, a pretty conclusive proof that time and accident do much to bring these cases to a favorable termination or to death; and I have no reason to think that many of these cases end in the destruction of life. In 530 instances the patient had either never indulged in sexual intercourse, or he had more or less completely abandoned it, generally from inability to continue it. In a large number of instances, masturbation was admitted; in many cases it did not seem to have been carried to any considerable extent; in a majority of cases, it was said that the practice had been discontinued for months, or even years, before the existence of the present complaint. In 597 cases the discharges did not occur more frequently than twice a week. In a great majority of cases, they only happened at night, during dreams, and the patient was conscious of their occurrence. In 27 cases, discharges were now and then observed to occur, during the straining at stool, and in some instances, after the passage of urine; and in these cases I have observed that the mental depression is much greater than in others, because quacks paint the consequences of such discharges in much gloomier colors than those which are accompanied by orgasm. In 16 only of my cases had the patient ever suffered from gonorrhœa, in only one instance could I make out any connection between any skin disease and the discharges. In only one instance could I satisfy myself that the discharges were kept up by irritation excited in the rectum by ascarides. In only four instances could they be referred to venereal excesses.

It will be understood, then, that in my experience the so-called involuntary discharges have not been attended with such disastrous results as in that of Lallemand. When I have witnessed these injurious results, I have been convinced that the discharges have usually been voluntary, that they have been more or less completely owing to masturbation, which the patient continued to practise. These I have found the most difficult to manage, for neither lunar caustic nor moral reflections will master the habit here, although all such cases in Lallemand's practice yielded to nitrate of silver. The most certain remedy appears to me to be sexual intercourse. I constantly tell patients that if the habit of masturbation be continued, they had better not submit to treatment, for it will be of no avail.

I have not observed either the almost certain good effects or the after-trouble to which Lallemand alludes when speaking of the application of lunar caustic; at the same time I have no doubt that the remedy is a valuable one.

It is singular how uniform is the description of symptoms given by patients in these cases. There are few who do not complain of loss of strength, loss of memory, and confusion of mind; there are many who complain of pains in the loins and palpitation of the heart; but in a very few cases are these complaints not more imaginary than real. The alleged loss of strength rarely interferes with the ability to perform ordinary duties. The loss of memory is real enough, but it is simply that the pre-occupation is so complete that nothing but the circumstance of the malady makes any impression on the mind; and the palpitation of the heart, unless under nervous excitement, is by no means of common occurrence.

My own experience has convinced me that the only certain means of relief in most cases is to be found in moderate sexual intercouse; it usually puts an end to masturbation, and the activity of the organs is most certainly mitigated by these means. I always feel a difficulty in recommending the remedy, because I cannot reconcile it to my conscience to advise a course of profligacy; and therefore I advise patients to mary, but, as may be supposed, a very common answer is, that it is inconvenient. It is never prudent, where a man alleges that his sexual energy is lost, to advise experimental conection, because, with the misgivings in his mind, he will most certainly fail and we shall in this way only add to his distress. If it be tried at all, some permanent connection should be formed, and he should be prepared to expect many failures, but a single success occurs, and the phantom which haunted his mind is at once dissipated.

In most of the cases, however, which have come in my way, sexual connection has not been attempted, and, either from fancied inability to accomplish it, or from an objection on other grounds, it will not be attempted. These cases are difficult to manage, and in all probability the discharge will persist in spite of all that may be done; and all that remains for us is to endeavor to convince the patient that when they do not occur oftener than twice a week years may pass without any weakening influence on the constitution being exerted by them. Even when all evil habits have ceased, that is to say, when masturbation is no longer practised, and lascivious images are carefully and completely excluded from the mind, these discharges will often persist for an indefinite time, apparently by virtue of the activity which has been set up in the organs, and which is long maintained by habit.

There is yet another class of patients who, although they suffer occasionally from involuntary discharges, complain principally of the too rapid ejaculation which occurs when conection is attempted, happening often before complete erection takes place. For the most part I have found such persons to posses very excitable temperaments, and they make these attempts unfrequently, although a species of sexual excitement is kept up. Again, in these cases the remedy is to be found in a regular and not too frequent relief to tension which is maintained in these organs. I have rarely known a case where such a plan has been faithfully followed without complete relief; but I have, in such cases, occasionally observed the good effects of cauterization, which, in them, seems to act by lessening irritability.

Respectable practitioners often do harm by exciting hopes of amendment or cure from the employment of tonics or stimulants, such as various preparations of iron and cantharides; they are very rarely of any use, and after persisting in such means, often for months, the patient is doomed to disappointment and increased despondency. The truth is, it is not debility which we have to do with, in most of these cases, but an increased activity of the secreting organs, and relief is most certainly obtained by periodically relieving the seminal vesicles from the distension to which they are subject.

The cases which are improved by local applications, whether of lunar caustic or any less energetic agent, are a small minority. In the early part of my experience, and following in the train of Lallemand, I applied lunar caustic to the urethra in most cases, and in many with seeming benefit to the patient; in some with complete relief to the symptoms, but in others the discharges continued unchanged.

The rule upon which I then acted I now follow; that is to say, I do not usually employ lunar caustic when there is no other indication of morbid action than is furnished by the occurrence of these discharges. Where there are increased sensibility, and chronic discharges, I often apply caustic upon the portion of the canal which appears to be the seat of morbid action, and often with great success. Where there is contraction of the canal of the uretha, I endeavor to overcome it by the use of dilating bodies; and when the discharges are kept up (which is in my experience very unfrequently the case) by such contractions, relief is sometimes obtained. But there are cases in which I have applied the lunar caustic, although there was no reason to believe in the existence of any morbid action on the mucous surface of the uretha. The truth is, that many persons present themselves under profound despondency. Many means have been employed and have failed; it is soon evident that they are not content to take advice only, but they are extremely anxious to have something done; and I have on many occasions applied caustic, because the patient had great faith in it, and would not be satisfied unless it were employed. In such cases I have first endeavored to dissipate from the patient's mind the fears by which he was beset. I have applied the remedy, and recommended the patient not to expect any very decided relief for two or three months. I have had no hesitation in applying caustic under such circumstances, because, with all my experience of it, I have never known any mischief to follow its use, except in two instances, where there was retention of urine in the evening of the day.

Whenever I apply caustic, I seek to determine a discharge which persists for twenty-four or forty-eight hours; if that effect is not produced, the full effect of the remedy is not obtained. If there be reason to think that a chronic discharge is kept up by inflammation or by strictures, and that the spermatic discharges are dependent thereupon, they must be got rid of before we can hope that the spermatic trouble will cease; and even when they are got rid of, habit may cause it to persist almost indefinitely.—*London Medical Gazette.*

